# The Effect of a Biodegradable Additive on the Mechanical and Morphological Properties of HDPE and LDPE Polymers

**DOI:** 10.3390/ma19194075

**Published:** 2026-09-23

**Authors:** Ömer Asal, Ayşe Selen Yücel

**Affiliations:** 1 Department of Manufacturing Engineering, Technology Faculty, Gazi University, 06500 Ankara, Türkiye; 2 Graduate School of Natural and Applied Sciences, Gazi University, 06500 Ankara, Türkiye; ayseselenyucel@gmail.com

**Keywords:** polymers, oxo-degradable additive, mechanical properties of plastics

## Abstract

The long degradation time of commonly used plastic materials leads to serious environmental pollution. Low-density polyethylene (LDPE) and high-density polyethylene (HDPE) are among the most widely used polymers in industrial applications. In this study, a commercially available oxo-degradable additive (Addiflex-HES), designed to promote oxidative degradation of conventional polyethylene, was incorporated into LDPE and HDPE at varying ratios, and the effect of this additive on the mechanical behavior of the materials was investigated. Within the scope of the study, specimens containing 0%, 1%, 3%, and 5% additive have been prepared. Test specimens were produced using injection molding in accordance with standard tensile and impact test geometries. Tensile tests, impact tests (Charpy and Izod), and Vicat softening temperature tests were conducted. In addition, the surface morphologies of the specimens were examined using scanning electron microscopy (SEM). The experimental results were analyzed comparatively. The findings showed that the additive content significantly affected the mechanical properties of both LDPE and HDPE. The oxo-degradable additive used in this study reduced the mechanical performance of both materials. The results showed that the mechanical properties of the LDPE samples were more pronouncedly affected by the biodegradable additive than those of the HDPE samples.

## 1. Introduction

Polyethylene is one of the most widely used polymeric materials due to its low cost, ease of processing, chemical resistance, and favorable mechanical properties. Low-density polyethylene (LDPE) and high-density polyethylene (HDPE) are extensively utilized in packaging, consumer goods, and engineering applications. Global plastic production has increased dramatically over the last few decades, generating large quantities of plastic waste. Due to their resistance to natural degradation processes, conventional polyolefins such as polyethylene can persist in the environment for many years, contributing to terrestrial and marine pollution. Consequently, the accumulation of polyethylene waste due to its long degradation time has become a major environmental concern worldwide [1,2,3]. Determining essential features, such as the use of recycled materials, is the first design request for industrial parts. Thermoplastic materials are lightweight and reasonably priced, but they are not as rigid and durable as metallic materials. When choosing a product material, factors like weight, strength, and load capacity are crucial [4]. By using additives such as fillers in recycled materials like PET, PP, and PE, it is possible to achieve mechanical properties that are close to those of virgin materials [5].

In response to these challenges, biodegradable additives have been introduced into polymer systems to enhance environmental degradation. Nevertheless, incorporating such additives often leads to structural changes in the polymer matrix, which can negatively affect mechanical performance. The mechanical behavior of LDPE and HDPE differs significantly due to their molecular structures; LDPE exhibits higher ductility and elongation, whereas HDPE provides higher stiffness and strength due to its higher crystallinity [3,6]. The degree of crystallinity plays a major role in determining the mechanical performance of polyethylene materials. In general, increasing crystallinity improves stiffness, hardness, and tensile strength, whereas lower crystallinity increases flexibility and elongation at break. Because LDPE and HDPE have different crystalline structures, incorporating biodegradable additives may influence their mechanical behavior differently. Therefore, comparative investigations involving both polyethylene types are important for understanding the relationship between additive content and mechanical performance [7,8].

Previous studies have shown that incorporating biodegradable or natural fillers into polyethylene matrices can disrupt the polymer structure and reduce mechanical strength. In particular, weak interfacial interactions between the additive and polymer matrix are considered one of the main reasons for the deterioration of mechanical properties. Poor adhesion between phases leads to ineffective stress transfer, resulting in reduced tensile strength and increased brittleness [9,10]. Besides interfacial adhesion, the dispersion quality of additives within the polymer matrix is another critical factor affecting composite performance. Non-uniform distribution or agglomeration of additive particles may generate local stress concentration regions that facilitate crack initiation under mechanical loading. Consequently, achieving a homogeneous distribution of biodegradable additives is essential for preserving the structural integrity of polyethylene-based materials [11]. Recycled plastic, glass, and aseptic carton boxes can be combined to produce composite plastic materials. When the amount of glass powder increases, the density also increases, but the tensile strength decreases when producing these composites. Moreover, as the weight percentage of glass powder increases, swelling and water uptake decrease [12].

Furthermore, adding waste glass powder has increased the hardness values of the composite plastics. The waste glass powder size affects the mechanical properties of the composite. When the powder size is decreased, flexural modulus, tensile modulus, and elongation at break increase, while the tensile strength decreases slightly [13].

In addition, the distribution and compatibility of the additive within the polymer matrix play a critical role in determining both mechanical performance and degradation behavior. Studies on polyethylene-based composites have demonstrated that increasing additive content generally reduces tensile strength and elongation, while promoting structural heterogeneity and micro-void formation. These morphological changes significantly influence material performance [14,15,16]. Several studies have shown that incorporating biodegradable components into polyethylene matrices can lead to voids, phase discontinuities, and heterogeneous microstructures. Such structural irregularities often reduce the material’s load-bearing capacity and negatively affect its tensile and impact performance. However, biodegradable additives continue to attract considerable attention because they offer a practical approach for improving the environmental sustainability of conventional plastics while maintaining acceptable engineering properties [17,18].

Another important factor affecting the behavior of biodegradable polymer systems is aging. Environmental exposure, such as thermal and oxidative conditions, accelerates degradation mechanisms, including chain scission and oxidation. These processes lead to embrittlement and further reduction in mechanical properties. Several studies have reported that biodegradable additives can accelerate oxidative degradation, leading to earlier mechanical failure than in neat polyethylene [19,20,21].

Processing conditions also play a significant role in determining the final properties of polyethylene materials. Parameters such as melt temperature, injection pressure, cooling rate, and mold conditions can influence crystallinity, molecular orientation, and residual stress formation within the polymer structure. These microstructural changes directly affect tensile strength, impact resistance, and dimensional stability of molded products. Therefore, in addition to additive content, processing parameters should also be considered when evaluating the mechanical performance of polyethylene-based materials. Although numerous studies have investigated biodegradable additives in polyethylene systems, most studies have focused on a single polyethylene type or on specific biodegradable filler systems. Comparative investigations involving both LDPE and HDPE under similar processing conditions remain relatively limited. In addition, many studies primarily focus on degradation behavior, whereas comprehensive evaluations of tensile, impact, and morphological properties at different additive concentrations are less frequently reported. Therefore, further research is required to better understand how biodegradable additives influence the mechanical performance of different polyethylene materials [22,23].

Biodegradation and oxidative degradation are two different processes. Abiotic chemical processes, such as oxidation-induced chain scission, are involved in oxidative degradation. These reactions can change the mechanical and morphological characteristics of the polymer and reduce its molecular weight. On the other hand, under the right circumstances, biodegradation is the biological transformation of polymeric materials or their breakdown products by microorganisms into environmentally significant end products such as CO_2_, H_2_O, and biomass. The oxidative degradation step and the subsequent biodegradation stage are clearly distinguished by ASTM D6954-24 [24], which also offers a framework for their independent assessment.

Numerous studies have focused on increasing the degradation rate of plastics by incorporating oxo-degradable additives in recent years. However, studies investigating the effects of such additives on the mechanical behavior of plastic materials remain limited. In this context, the present study aims to investigate the mechanical and morphological behavior of LDPE and HDPE materials containing different ratios of an oxo-degradable additive (Addiflex-HES). The materials were produced under controlled injection molding conditions, and their mechanical properties were evaluated using tensile and impact tests. Additionally, morphological analyses were performed to better understand the structural changes induced by the additive.

This study contributes to the literature by (i) providing a comparative evaluation of LDPE and HDPE under the same additive system, (ii) examining the effect of additive content over a wide concentration range, and (iii) analyzing both additive-containing and additive-free systems in terms of mechanical performance. The findings of this study aim to support the development of sustainable polymer materials by balancing environmental degradability and mechanical reliability.

## 2. Materials and Methods

In this study, a commercially available oxo-degradable additive, Addiflex-HES, was used to modify polyethylene-based materials. This additive is designed to accelerate the environmental degradation of conventional thermoplastics via oxidative mechanisms.

Low-density polyethylene (LDPE, PETILEN 122-19T) [25] and high-density polyethylene (HDPE, I668 Eltex) [26] were used as matrix materials. LDPE consists of branched hydrocarbon chains (–CH2–CH2–)n, resulting in lower crystallinity and higher ductility, whereas HDPE has a more linear structure with higher crystallinity, providing greater stiffness and strength.

Four distinct weight fractions of the oxo-degradable additives were added to the LDPE and HDPE matrices: 0%, 1%, 3%, and 5%. Samples were prepared at various injection temperatures and pressures and divided into discrete groups to assess the combined impact of processing parameters and additive content.

Test specimens were produced by injection molding. Prior to production, the additive was accurately weighed using a precision balance (0.1 mg sensitivity) and pre-packaged to ensure repeatability and consistency. The polymer granules and additives were manually mixed to achieve approximate homogeneity and then fed into the injection molding machine.

The injection molding process was carried out under controlled temperature and pressure conditions. Injection temperatures ranged from 160 to 180 °C for LDPE, while higher temperatures (190–210 °C) were used for HDPE due to its higher melting point. Injection pressures of 6 MPa, 8 MPa, and 10 MPa were applied during the molding of the specimens for the experimental studies. The mold temperature was maintained at approximately 60 °C by applying cooling to the injection mold.

The cooling time was calculated and applied according to the following formula:

(1) $$t_{c}=\frac{s^{2}}{\pi^{2}\alpha }ln\left[\frac{8\;(Tm-Tc)}{\pi^{2}(Te-Tc)}\right]$$

where t_c_ is cooling time, s is half-thickness of the part, α is thermal diffusivity, *Tm* is melt temperature, *Tc* is mold temperature, and *Te* is ejection temperature.

The temperature of the samples taken from the mold was approximately 90 °C. To prevent defects such as shrinkage and void formation, cooling conditions and holding times were optimized. Samples were rapidly cooled in a water bath (~15 °C) immediately after molding to improve dimensional stability.

A mold with standard specimen cavities for ISO 527-1 [27] tensile test and ISO 179-1 [28] and ISO 180-1 [29] impact test specimens was used in the experimental studies.

Tensile tests were conducted in accordance with ISO 527-1 to evaluate the mechanical behavior of the specimens produced. For each experimental condition, three specimens were tested, and average values were considered. The crosshead speed was arranged as 5 mm/min in the tensile tests.

Charpy and Izod impact tests were used to determine impact properties. Given their distinct mechanical characteristics, HDPE specimens were tested using the Charpy method (ISO 179-1), and LDPE specimens were tested using the Izod method (ISO 180-1).

Additionally, Vicat softening temperature tests were conducted in accordance with ASTM D1525 [30] to evaluate the thermal softening behavior of the materials.

In addition to mechanical tests, hardness measurements were performed to evaluate the surface resistance of the produced specimens. A Shore-D hardness tester was used to measure hardness. Five repetitions of each test were conducted using a force of 5 kgf.

The surface morphology of the specimens was analyzed using scanning electron microscopy (SEM). Prior to imaging, samples were coated with a thin conductive layer using a sputter coater to prevent charging.

SEM images were obtained at appropriate accelerating voltages and magnifications. For each specimen, multiple images were taken to ensure reliable morphological evaluation.

## 3. Results

### 3.1. Tensile Test Results

The tensile behavior of LDPE specimens under different processing conditions is presented in Figure 1 and Figure 2. As shown in Figure 1, elongation at break increased with increasing injection temperature from 160 °C to 180 °C for neat LDPE. This behavior is attributed to improved melt flow, enhanced chain mobility, and a more homogeneous polymer structure at higher temperatures, which facilitates plastic deformation. In contrast, the specimen produced at a lower temperature (160 °C) exhibited relatively higher stress but lower elongation, indicating a more rigid response due to limited molecular mobility.

When the effect of injection pressure was evaluated at 180 °C (Figure 2), elongation increased from 6 MPa to 8 MPa but decreased at 10 MPa. The observed variation may be related to changes in melt flow, molecular orientation, cooling history, and residual stress development associated with the processing conditions [31]. Although changes in crystallinity or molecular packing may contribute to the observed mechanical response, these mechanisms were not directly characterized in the present study by techniques such as differential scanning calorimetry (DSC) or X-ray diffraction (XRD).

In contrast to neat LDPE, elongation decreased with increasing temperature in LDPE containing a 1% oxo-degradable additive. Although higher temperatures slightly increased stress levels, ductility was reduced. This behavior is associated with the additive, which restricts chain mobility and promotes the formation of heterogeneous regions within the polymer matrix. In addition, longer cooling times at higher temperatures may promote the formation of a more crystalline region, further limiting elongation. Previous studies have reported that elongation at break is highly sensitive to thermal effects in oxo-biodegradable polyethylene systems [32,33,34,35].

As shown in Figure 3, elongation initially increases with temperature from 190 °C to 200 °C, then decreases slightly at 210 °C. This behavior indicates that moderate processing temperatures enhance chain mobility and promote molecular rearrangement, whereas excessive temperatures may lead to structural instability or localized degradation. However, this improvement in ductility remains limited compared to LDPE due to HDPE’s inherently high crystallinity, which restricts chain mobility even at elevated temperatures. Similar findings have been reported in the literature, where HDPE maintains its stiffness despite changes in processing parameters [36].

The pressure–elongation behavior (Figure 4) shows a decreasing trend with increasing injection pressure. This behavior may be associated with changes in molecular orientation, residual stresses, cooling history, and the resulting morphology of the injection-molded specimens. An increase in molecular packing or crystallinity could potentially contribute to the reduction in deformation capability [36]. However, crystallinity was not directly measured in this study.

The comparative stress–strain curves (Figure 5) further support these observations. Neat LDPE exhibited a wide plastic deformation region, characteristic of highly ductile behavior. In contrast, additive-containing specimens showed reduced elongation, lower strain at break, and a progressive narrowing of the plastic region. At higher additive contents, the material exhibited abrupt fracture, indicating a clear transition from ductile to brittle behavior.

Overall, increasing the oxo-degradable additive content significantly reduced elongation and narrowed the plastic deformation region in LDPE specimens. While processing parameters such as temperature and pressure influenced the mechanical response to some extent, the additive content was the dominant factor governing tensile behavior. Changes in material structure, interfacial bonding, and additive distribution directly influenced deformation behavior and failure mechanisms. While neat LDPE exhibited highly ductile behavior, additive-containing specimens showed a transition toward brittle behavior. At higher additive contents, the additive acted as a source of defects rather than a reinforcing phase, leading to early fracture [35].

The tensile behavior of HDPE specimens under varying processing conditions is presented in Figure 6. Compared to LDPE, HDPE exhibits a more rigid mechanical response due to its higher crystallinity and more linear molecular structure. Accordingly, the stress–strain curves show higher strength but significantly lower elongation, indicating a more limited capacity for plastic deformation.

For higher additive contents, elongation values decrease significantly, and the mechanical response becomes increasingly brittle. Although slight variations with temperature and pressure are observed, the overall behavior is dominated by the additive. The stress–strain curves (Figure 6) show a progressive reduction in the plastic deformation region and earlier fracture points as the additive content increases. At additive contents of 3% and 5%, the material exhibits clearly brittle behavior, with minimal elongation and abrupt failure. This behavior may be associated with changes in the polymer microstructure and the formation of local discontinuities introduced by the additive. The presence of additive particles disrupts intermolecular bonding and creates structural discontinuities within the matrix, thereby significantly reducing load transfer efficiency and promoting early crack initiation. Literature reports confirm that increasing the content of oxo-degradable additives reduces ductility and accelerates degradation in polyethylene systems [37].

Overall, HDPE specimens exhibit a transition from ductile to brittle behavior as additive content increases. While processing parameters such as temperature and pressure influence mechanical behavior to some extent, the additive concentration remains the primary factor controlling tensile performance. These findings indicate that additive–matrix interactions, structural homogeneity, and crystallinity are key parameters governing the overall mechanical behavior of HDPE systems [38].

### 3.2. Impact Test Results

The impact test results of LDPE and HDPE specimens with varying additive contents and processing conditions are presented in Figure 7 and Figure 8. The results clearly demonstrate that both polymer type and the content of the oxo-degradable additive significantly influence the impact resistance of the materials.

For neat LDPE specimens, relatively high impact strength values were obtained, indicating a ductile behavior under sudden loading conditions. The impact energy decreased slightly with increasing additive rates and pressure, suggesting that higher processing parameters promote increased crystallinity and reduced energy absorption capacity. Nevertheless, the overall response remained ductile due to LDPE’s flexible molecular structure.

A similar trend was observed for HDPE specimens. Neat HDPE specimens exhibited lower impact strength compared to LDPE due to their more rigid and highly crystalline structure. The impact energy decreased with increasing additive rates and pressure, indicating reduced energy absorption capability due to increased molecular packing. These findings indicate that even low additive content significantly affects HDPE by reducing its already limited ductility and promoting earlier crack initiation.

Overall, increasing the additive content led to a clear transition from ductile to brittle fracture behavior in both LDPE and HDPE specimens. The reduction in impact resistance can be attributed to the formation of structural discontinuities, weak interfacial bonding, and stress concentration points within the polymer matrix. These factors reduce load transfer efficiency and facilitate crack initiation and propagation under impact loading. Consequently, while oxo-degradable additives enhance environmental degradability, they significantly reduce impact performance and promote brittle failure mechanisms [35].

### 3.3. Thermal Softening Results

The thermal softening behavior of LDPE and HDPE specimens was evaluated using Vicat softening temperature measurements in accordance with ASTM D1525, and the results are summarized in Table 1. The findings indicate that both polymer type and the content of the oxo-degradable additive influence the materials’ thermal softening behavior.

Neat HDPE specimens exhibited significantly higher Vicat softening temperatures (~132–133 °C) than LDPE (~101–102 °C). This difference indicates that HDPE retained its dimensional resistance to the specified Vicat deformation at a higher temperature than LDPE under the test conditions. The difference is consistent with the different molecular structures and mechanical characteristics of the two polyethylene grades.

With the incorporation of an oxo-degradable additive, only slight variations in Vicat softening temperature were observed for both polymer types. In LDPE specimens, the Vicat temperature values fluctuated within a narrow range (100.90–101.90 °C), showing no clear decreasing trend as the additive content increased. This result indicates that the additive introduces minor structural changes but does not significantly affect the thermal softening behavior of LDPE.

For HDPE specimens, the value decreased at 1% additive content, then increased slightly at 3% and 5%. These variations suggest that the additive had a limited effect on the Vicat softening behavior under the investigated conditions.

The minor variations observed in Vicat softening temperature can be attributed to localized structural changes and slight weakening of intermolecular interactions caused by the additive. The presence of an oxo-degradable additive may increase chain mobility; however, this effect is not strong enough to significantly alter the softening temperature of the polymer matrix.

Overall, the results demonstrate that incorporating an oxo-degradable additive has a limited effect on thermal softening behavior, compared to its pronounced influence on mechanical properties. Although slight variations in Vicat softening temperature are observed, both LDPE and HDPE retain their general thermal characteristics, suggesting they may still be suitable for applications requiring moderate thermal softening.

### 3.4. SEM Analysis

Scanning electron microscopy (SEM) was used to evaluate morphological changes in LDPE and HDPE samples with varying contents of oxo-degradable additives and to examine the structure of the additives. The SEM image of the oxo-degradable material (Figure 9) shows a heterogeneous morphology with rough surfaces, void-like structures, and broad undulating regions, indicating a non-uniform phase structure that may limit compatibility with polymer matrices.

For neat LDPE specimens, the surface morphology appeared relatively smooth and homogeneous (Figure 10). This indicates a uniform polymer matrix with good structural integrity. With the addition of an oxo-degradable additive, noticeable changes in surface morphology were observed. At a low additive content (1%), a fine, dense distribution was observed, along with small voids and slight surface irregularities, indicating the initial disruption of the polymer matrix. At higher additive contents (3% and 5%), the surface became increasingly heterogeneous, with more pronounced voids, broad wavy regions, and phase separations. These structural discontinuities suggest poor interfacial compatibility between the additive and the polymer matrix. Weak interfacial bonding or phase incompatibility can lead to defect formation and reduced mechanical performance [38].

Similarly, HDPE specimens showed a relatively smooth, compact structure in the absence of an additive (Figure 11). At 1% additive content, small circular voids and oriented linear features were observed, indicating the onset of microstructural disruption. As the additive content increased to 3% and 5%, more pronounced undulating regions and void-like structures were observed, reflecting increased heterogeneity and reduced matrix continuity.

Overall, increasing the additive content led to the formation of microstructural defects, such as voids and phase-separated regions, in both LDPE and HDPE. These defects indicate weak interfacial bonding and act as stress concentration points, explaining the reduction in mechanical performance and the transition toward more brittle behavior.

### 3.5. Hardness Measurements

The results indicated that hardness varied with both polymer type and oxo-degradable additive content. In general, HDPE specimens exhibited higher hardness than LDPE, attributed to their higher crystallinity and more rigid molecular structure. The hardness values were measured using a Shore-D hardness tester. For HDPE specimens, the average hardness values decreased as the additive content increased. The neat HDPE sample (no additives) exhibited the highest hardness values (~95.5–99), while the 1% additive sample showed slightly lower values (~92–95). A more noticeable decrease was observed with the 3% additive (~89.5–93.5) and the 5% additive (~84.5–86). This trend indicates that increasing additive content disrupts the crystalline structure and reduces intermolecular interactions, leading to a softer material. Similarly, LDPE specimens showed a slight decrease in hardness as additive content increased. The neat LDPE sample (0% additive) exhibited values of ~90.5–92.5, while the 1% additive sample showed slightly lower values (~87–91.5). At higher additive contents, hardness values fluctuated but generally remained lower than those of the neat material. This behavior suggests that the oxo-degradable additive introduces structural irregularities within the polymer matrix, reducing surface resistance. The hardness values for the HDPE and LDPE samples at different additive concentrations are shown in Figure 12, along with their standard deviations.

Additionally, the oxo-degradable additive itself exhibited hardness values (~97–97.5), which are comparable to or higher than those of the polymer matrices. Despite the additive’s relatively high intrinsic hardness, its incorporation into the polymer matrix reduced the overall hardness. This situation indicates that the additive does not act as a reinforcing phase but instead creates weak interfacial regions and structural discontinuities. Overall, the results demonstrate that increasing the oxo-degradable additive content reduces hardness in both LDPE and HDPE systems. This behavior is consistent with tensile and impact test results, which show that the additive reduced mechanical performance by weakening intermolecular interactions and promoting structural heterogeneity.

### 3.6. Statistical Analysis

The effects of additive rate and injection pressure on the elongation behavior of LDPE and HDPE were evaluated using analysis of variance (ANOVA). The statistical results for LDPE are presented in Table 2. As shown in Table 2, the additive rate had a statistically significant effect on the elongation of LDPE (F = 33.88, *p* < 0.001), whereas the effect of pressure was not statistically significant (F = 0.75, *p* = 0.512). The additive rate accounted for 93.13% of the total variation in elongation, while pressure contributed only 1.38%. These results indicate that the variation in LDPE elongation was predominantly associated with changes in additive content rather than injection pressure.

The main effects and interaction plots presented in Figure 13 further illustrate these trends. The main effects plot in Figure 13a shows a pronounced change in mean elongation with additive rate, whereas the variation associated with pressure is comparatively limited. The interaction plot in Figure 13b provides an additional visualization of the combined effects of the experimental factors. The relatively dominant effect of additive rate observed in the statistical analysis is consistent with the trends shown in these plots. The regression model also demonstrated strong explanatory power, with R^2^ = 94.50%, adjusted R^2^ = 89.92%, and predicted R^2^ = 78.01%, indicating that most of the variation in LDPE elongation was explained by the model.

The effects of additive rate and pressure on E Impact were also evaluated using ANOVA. For LDPE, the results are presented in Table 3. Both the additive rate and pressure were found to have statistically significant effects on E Impact. The additive rate had an F-value of 27.18 (*p* = 0.001) and accounted for 74.04% of the total variation. Pressure was also statistically significant, with F = 11.29 and *p* = 0.009, accounting for 20.51% of the variation. Therefore, although the additive rate was the dominant factor affecting the E Impact response of LDPE, pressure also contributed considerably to the observed variability.

The effects of the individual factors are shown in Figure 14a. The main effects plot demonstrates a clear change in E Impact with additive rate, and a measurable variation with pressure. The interaction plot in Figure 14b illustrates the combined response of E Impact to the two experimental factors. These graphical observations are consistent with the ANOVA results in Table 3, confirming that both factors should be considered when evaluating the impact performance of LDPE.

The statistical model for LDPE E Impact provided a high coefficient of determination (R^2^ = 94.55%) with an adjusted R^2^ of 90.01% and a predicted R^2^ of 78.21%. These values indicate that the model explains a substantial proportion of the variation in E Impact. However, the lower predicted R^2^ relative to the adjusted R^2^ suggests that the model’s predictive capability is somewhat lower than its explanatory capability and should therefore be interpreted within the investigated experimental range.

For HDPE, the ANOVA results are given in Table 4. The additive rate exhibited an even stronger effect on elongation than that observed for LDPE. Specifically, the additive rate was statistically significant (F = 349.16, *p* < 0.001) and accounted for 98.74% of the total variation. In contrast, pressure accounted for only 0.69% of the variation and was not statistically significant at the 95% confidence level (F = 3.66, *p* = 0.091). Thus, the elongation response of HDPE was predominantly governed by the additive rate under the processing conditions investigated.

This strong statistical contribution of the additive rate is also reflected in the main effects and interaction plots in Figure 15. As shown in Figure 15a, the mean elongation changes substantially with additive rate, while the effect of temperature/pressure is comparatively small. The interaction behavior shown in Figure 15b is consistent with the ANOVA results and further demonstrates the dominant role of the additive rate. The statistical model exhibited an excellent fit, with R^2^ = 99.43%, adjusted R^2^ = 98.96%, and predicted R^2^ = 97.74%. The substantially higher R^2^ values obtained for HDPE compared with LDPE indicate that the experimental factors considered in the model explain the elongation response of HDPE particularly well.

For HDPE, the corresponding ANOVA results are presented in Table 5. Similarly to LDPE, both the additive rate and pressure had statistically significant effects on E Impact. The additive rate was the dominant factor, accounting for 75.19% of the total variation, with F = 34.65 and *p* < 0.001. Pressure accounted for a further 20.47% of the variation and was also statistically significant (F = 14.15, *p* = 0.005). Therefore, in contrast to the elongation response, where pressure was not statistically significant for HDPE, pressure had a significant effect on the E Impact behavior of HDPE.

The trends obtained from the statistical analysis are also evident in Figure 16. The main effects plot in Figure 16a shows that E Impact varies considerably with additive rate and also changes with pressure. The interaction plot in Figure 16b illustrates the combined influence of these parameters. The agreement between the graphical trends in Figure 16 and the ANOVA results in Table 5 supports the statistical significance of both factors.

The HDPE E Impact model yielded R^2^ = 95.66%, adjusted R^2^ = 92.04%, and predicted R^2^ = 82.64%. These results demonstrate a strong overall model fit and indicate that the selected experimental factors explain most of the variation observed in the E Impact response.

A comparison of the ANOVA results demonstrates that the additive rate was the predominant factor affecting both elongation and E Impact for both polymer types. Its influence was particularly pronounced for elongation. The additive rate explained 93.13% and 98.74% of the variation in elongation for LDPE and HDPE, respectively. In contrast, the contribution of pressure was only 1.38% for LDPE and 0.69% for HDPE, and pressure was not statistically significant for either material.

The E Impact response exhibited a different sensitivity to the processing parameters. For LDPE, the additive rate and pressure explained 74.04% and 20.51% of the total variation, respectively. Correspondingly, for HDPE, these contributions were 75.19% and 20.47%, respectively. Therefore, pressure played a considerably more important role in determining E Impact than in determining elongation.

## 4. Conclusions

In this study, the effects of an oxo-degradable additive (Addiflex-HES) on the mechanical, thermal, and morphological properties of LDPE and HDPE were systematically investigated under different processing conditions.

The results clearly demonstrated that additive content is the dominant factor governing the material behavior in both polymer systems. Increasing additive concentration led to a significant reduction in tensile elongation and impact resistance, indicating a transition from ductile to brittle behavior. This effect was more pronounced in LDPE, which has inherently higher chain mobility and greater sensitivity to structural disruptions. In contrast, HDPE exhibited a more rigid response due to its higher crystallinity.

SEM observations revealed increased morphological heterogeneity and void-like features with increasing Addiflex-HES content. These features may be associated with differences in additive dispersion and local matrix–additive interactions and are consistent with the observed deterioration in mechanical performance.

In contrast to the mechanical properties, the Vicat softening temperature showed only minor variations as the Addiflex-HES content increased. HDPE exhibited higher Vicat softening temperatures than LDPE, while the additive produced no consistent or pronounced change in softening behavior under the conditions investigated. These results indicate that the additive had a limited effect on resistance to the specified Vicat deformation.

Although processing parameters such as temperature and pressure influenced the mechanical response to some extent, their effect remained secondary to that of the additive content. The results indicate that increasing the additive concentration reduces structural integrity and promotes brittle failure mechanisms under all processing conditions.

Overall, while the oxo-degradable additive enhances the environmental degradability of polyethylene-based materials, it significantly compromises mechanical performance. Therefore, optimizing additive concentration is critical to achieving a balance between degradability and structural reliability. These findings provide valuable insight for the design of sustainable polymer systems, particularly in applications where both environmental performance and mechanical integrity are required.

## Figures and Tables

**Figure 1:**
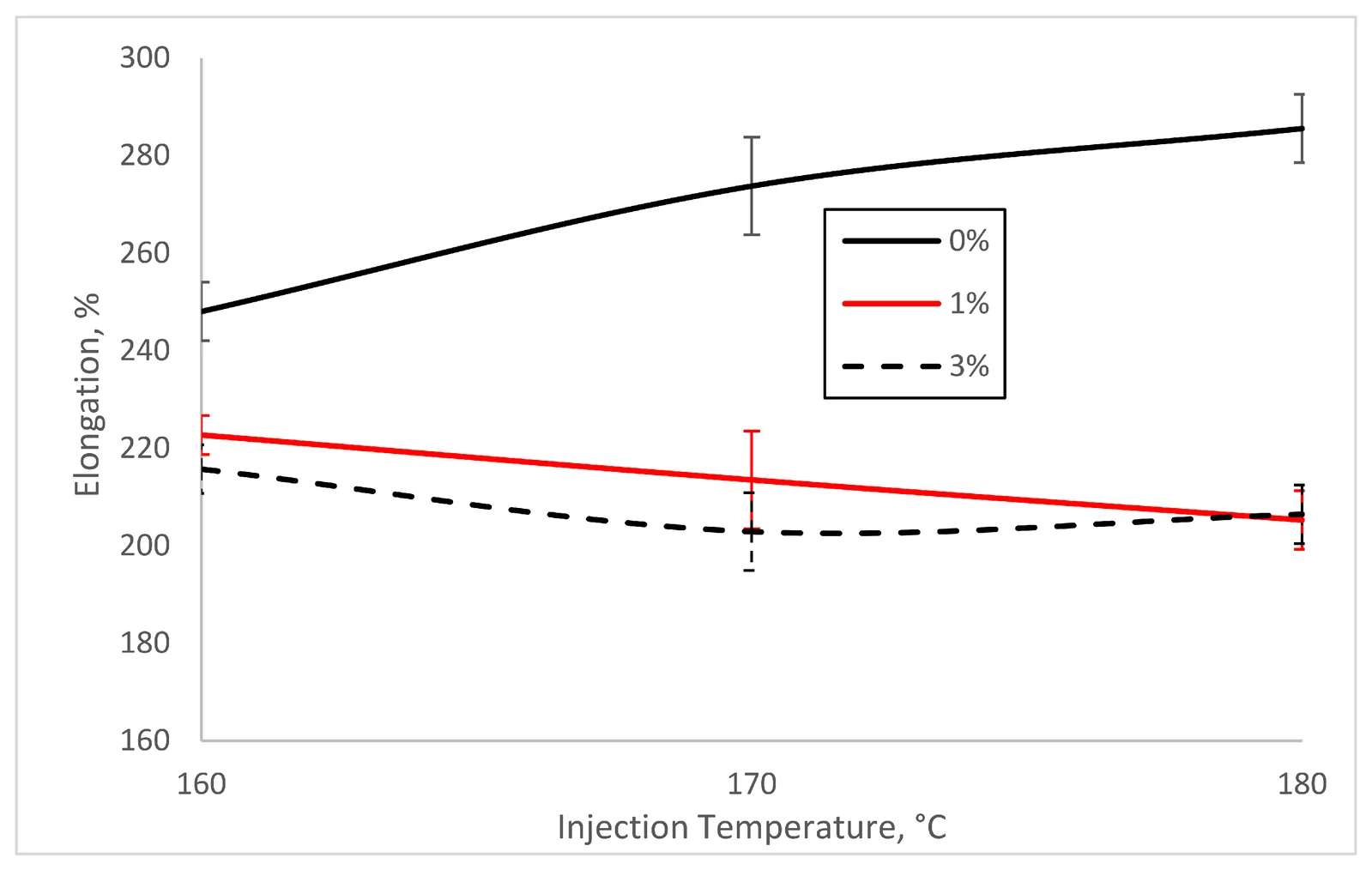
Temperature–elongation graph of LDPE according to the additive rates.

**Figure 2:**
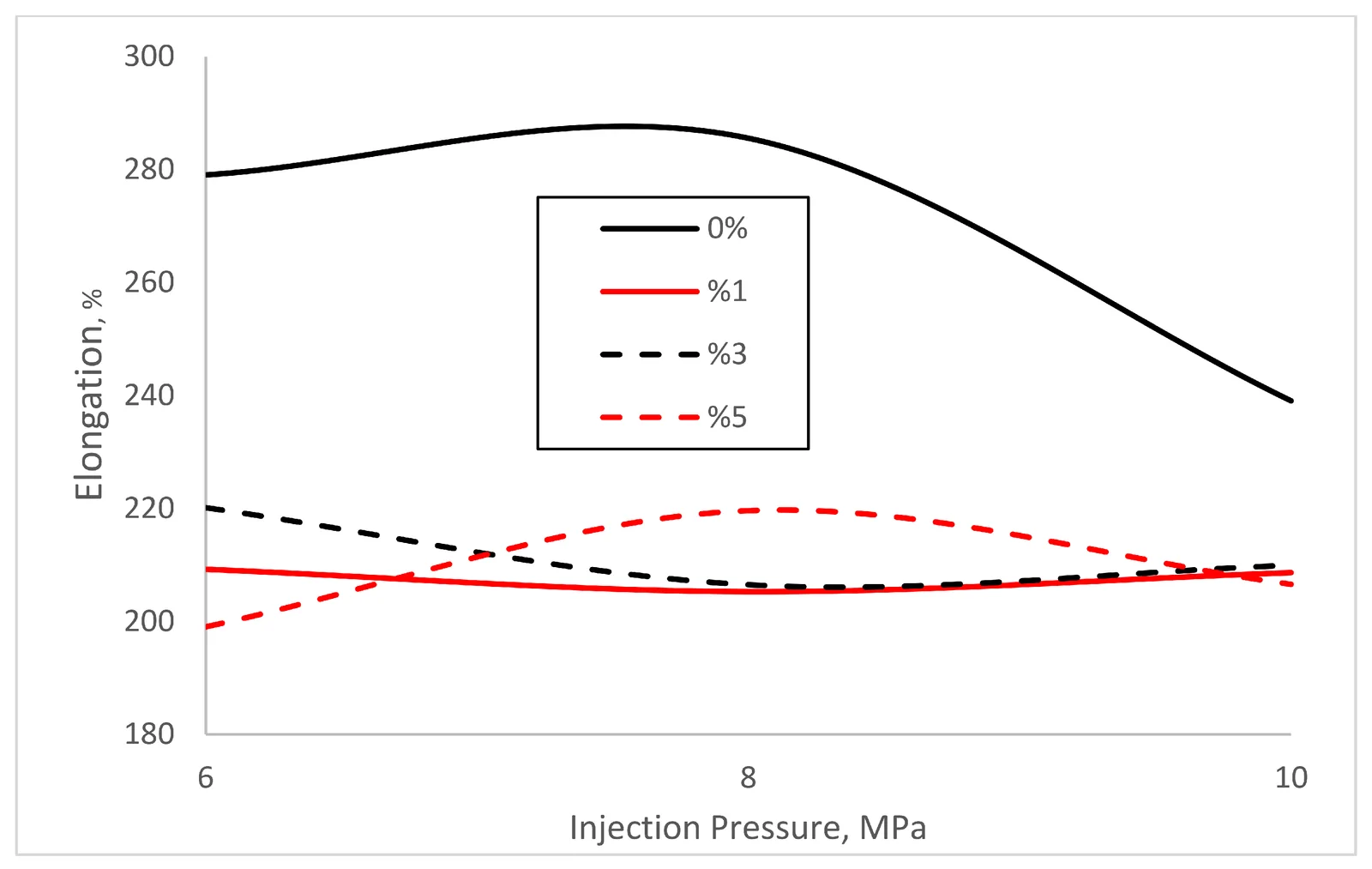
Injection pressure–elongation graph of LDPE according to the additive rates.

**Figure 3:**
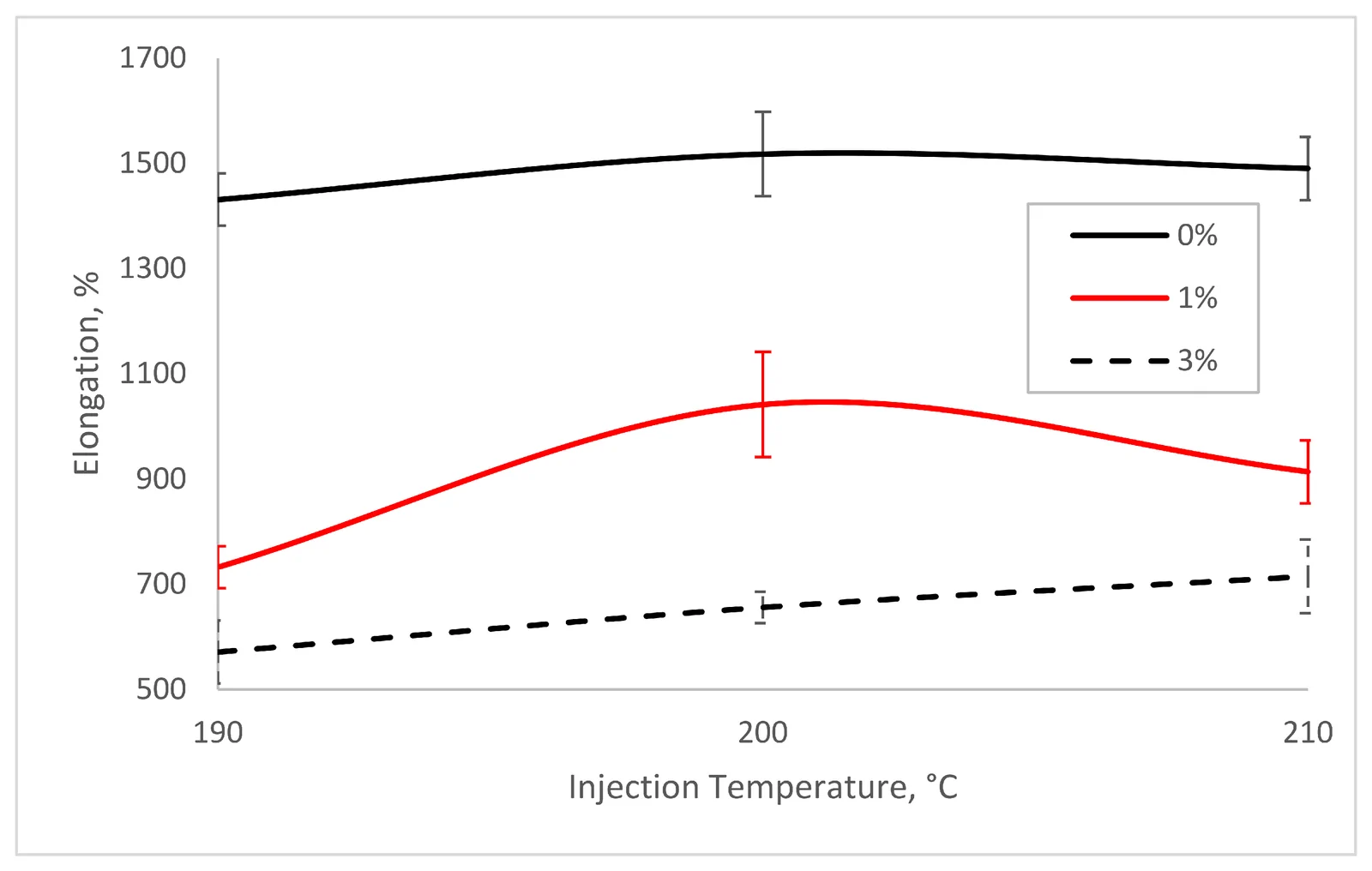
Temperature–elongation graph of HDPE according to the additive rates.

**Figure 4:**
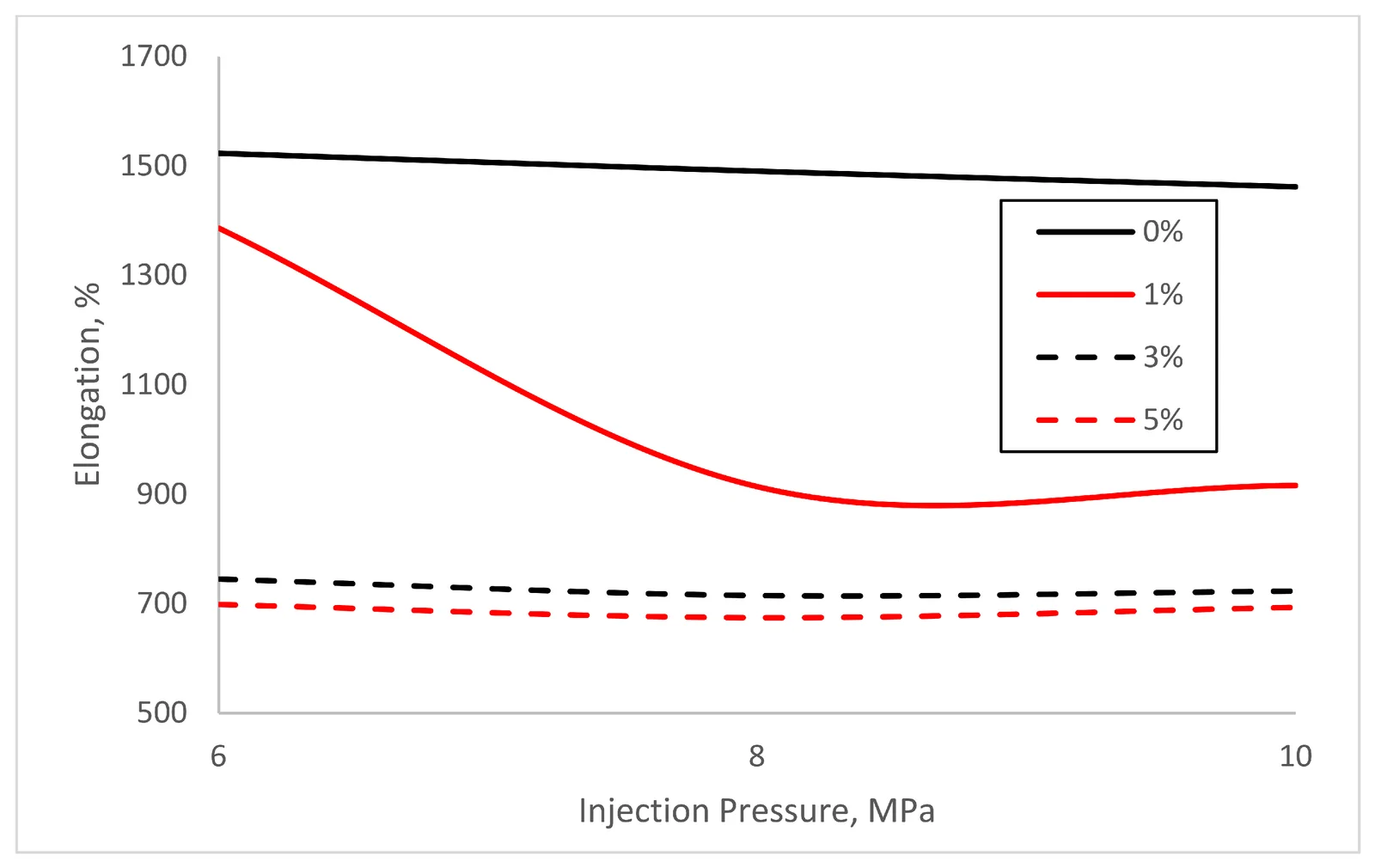
Injection pressure–elongation graph of HDPE according to the additive rates.

**Figure 5:**
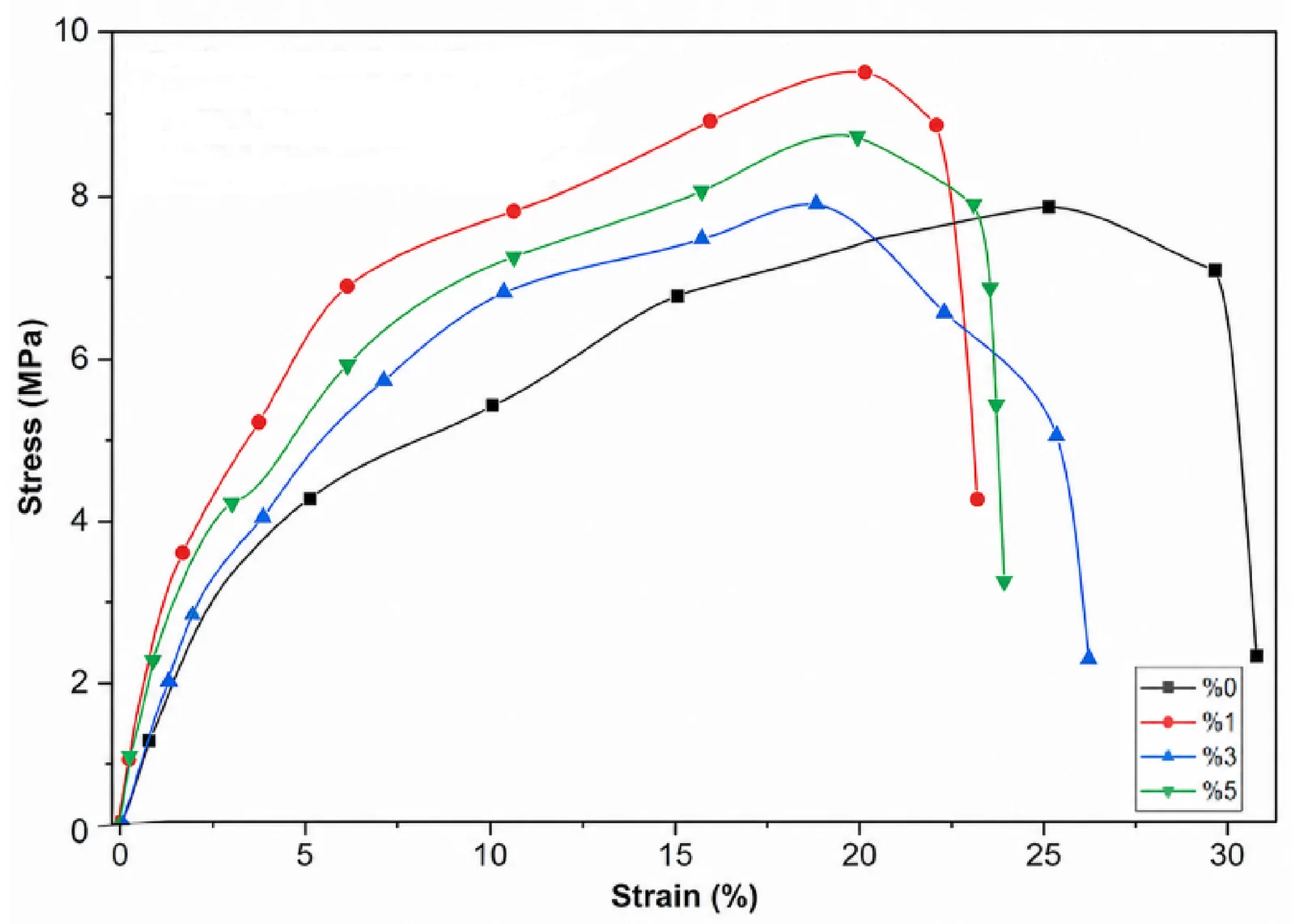
Stress–strain graph of LDPE according to the additive rates.

**Figure 6:**
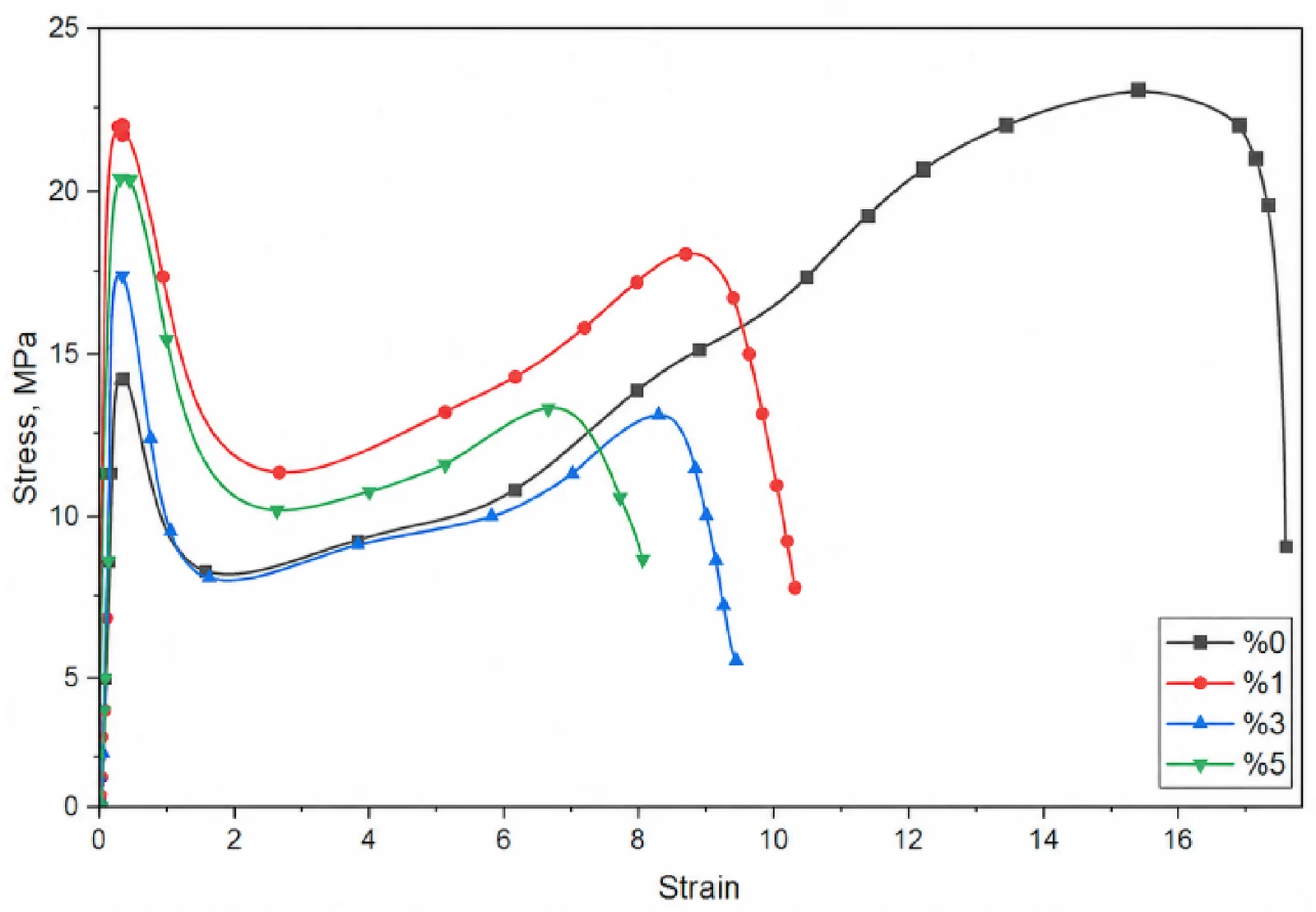
Stress–strain graph of HDPE according to the additive rates.

**Figure 7:**
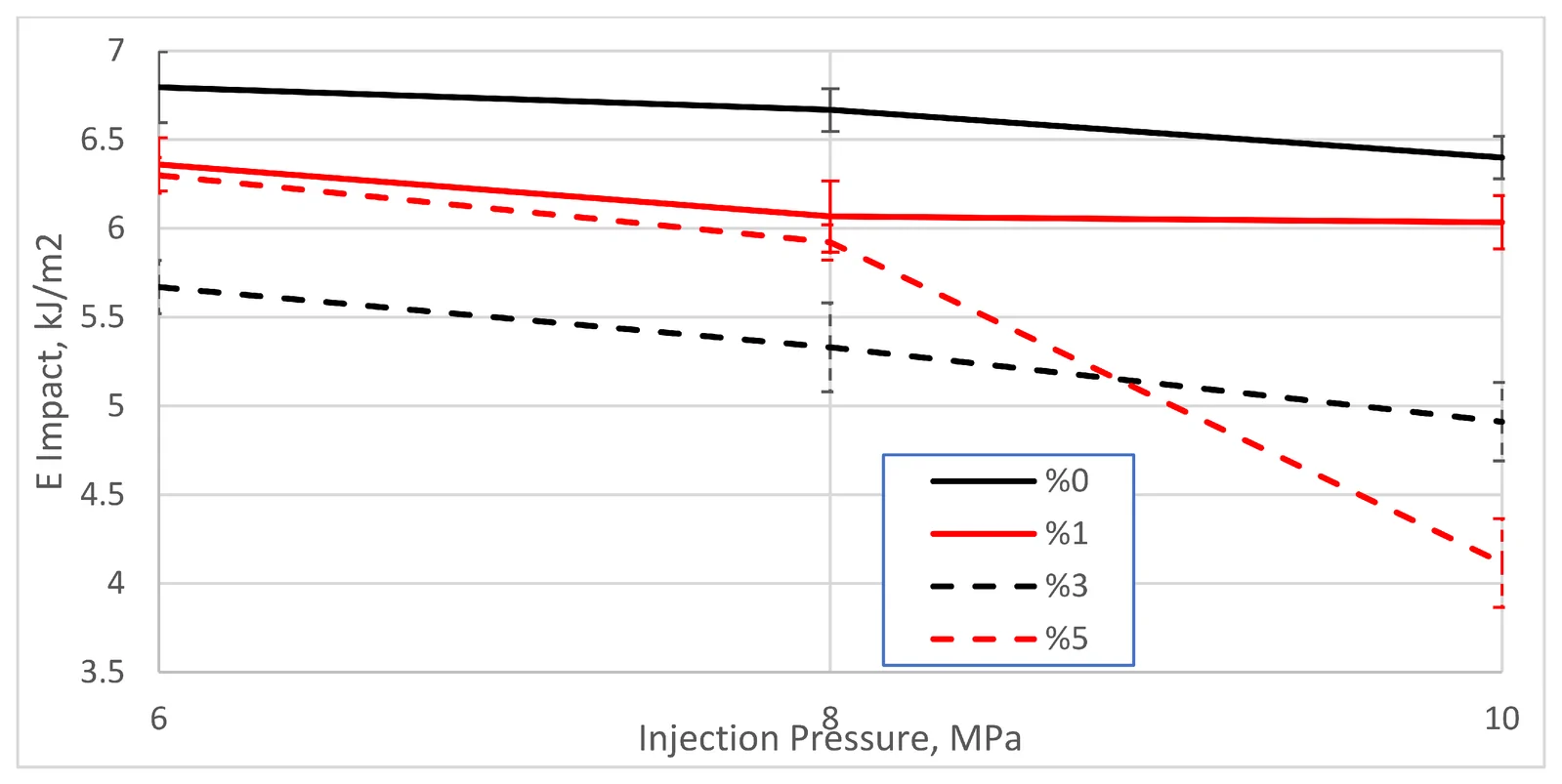
Impact energies of LDPE according to the additive rates and injection pressure.

**Figure 8:**
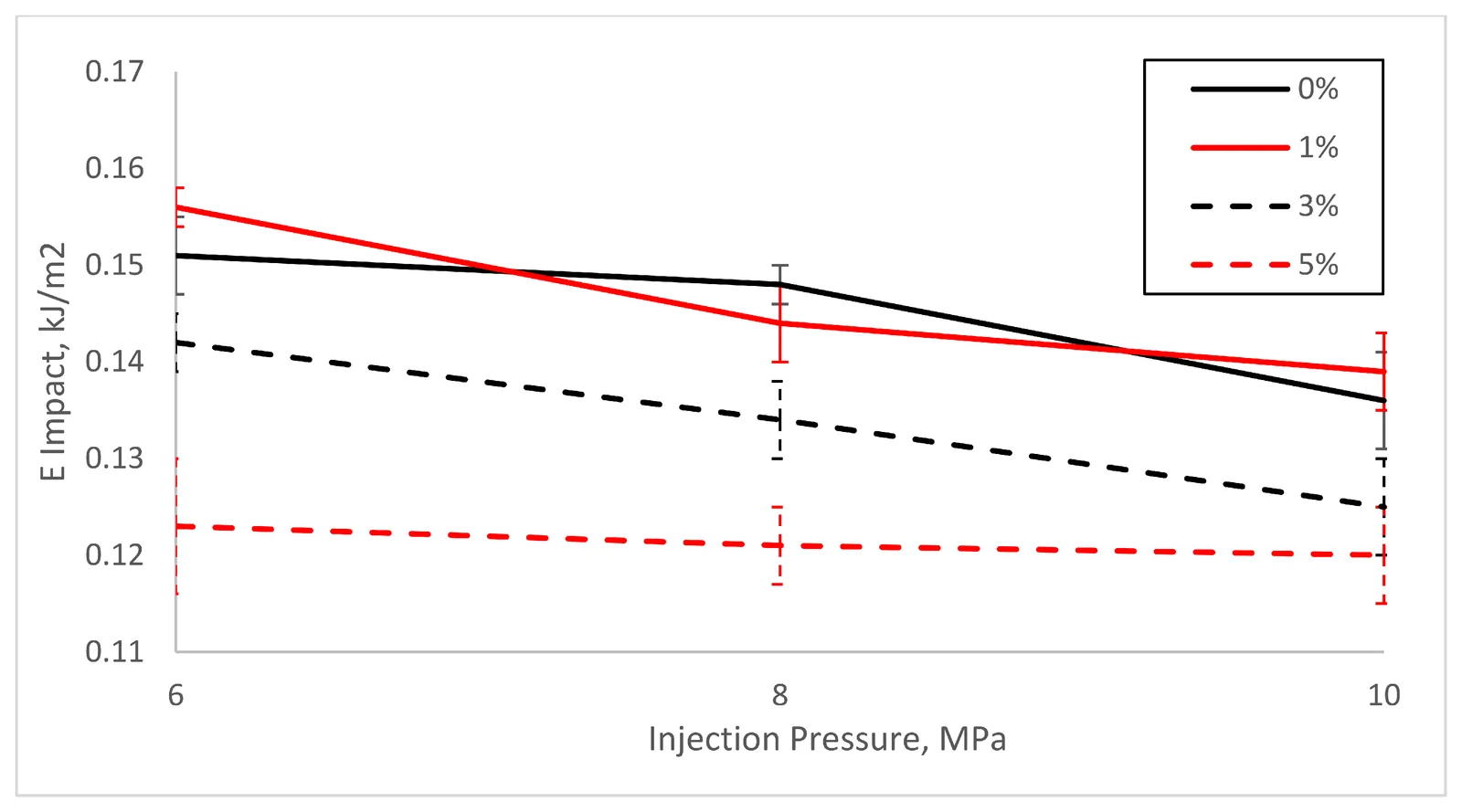
Impact energies of HDPE according to the additive rates and injection pressure.

**Figure 9:**
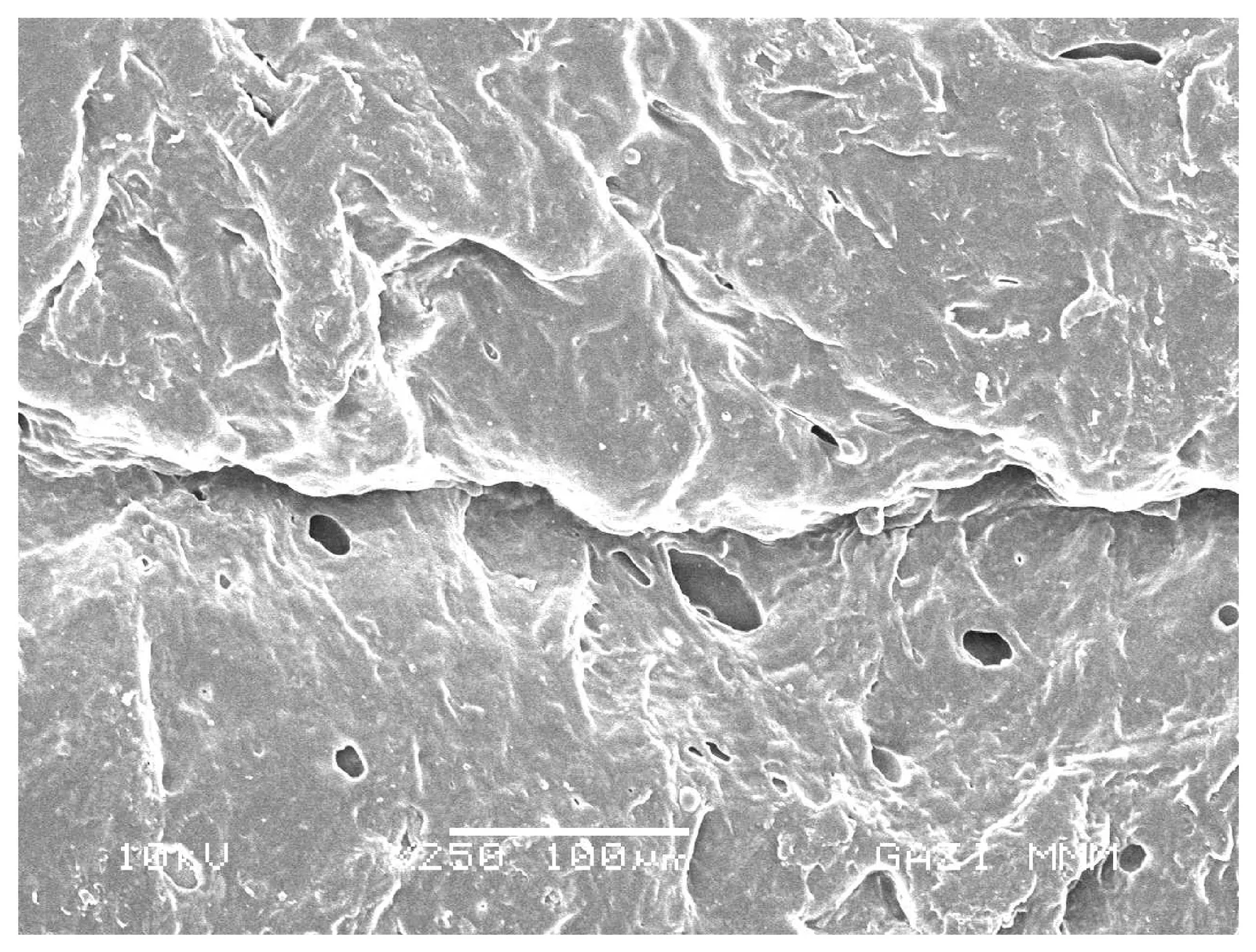
SEM image of the oxo-degradable material.

**Figure 10:**
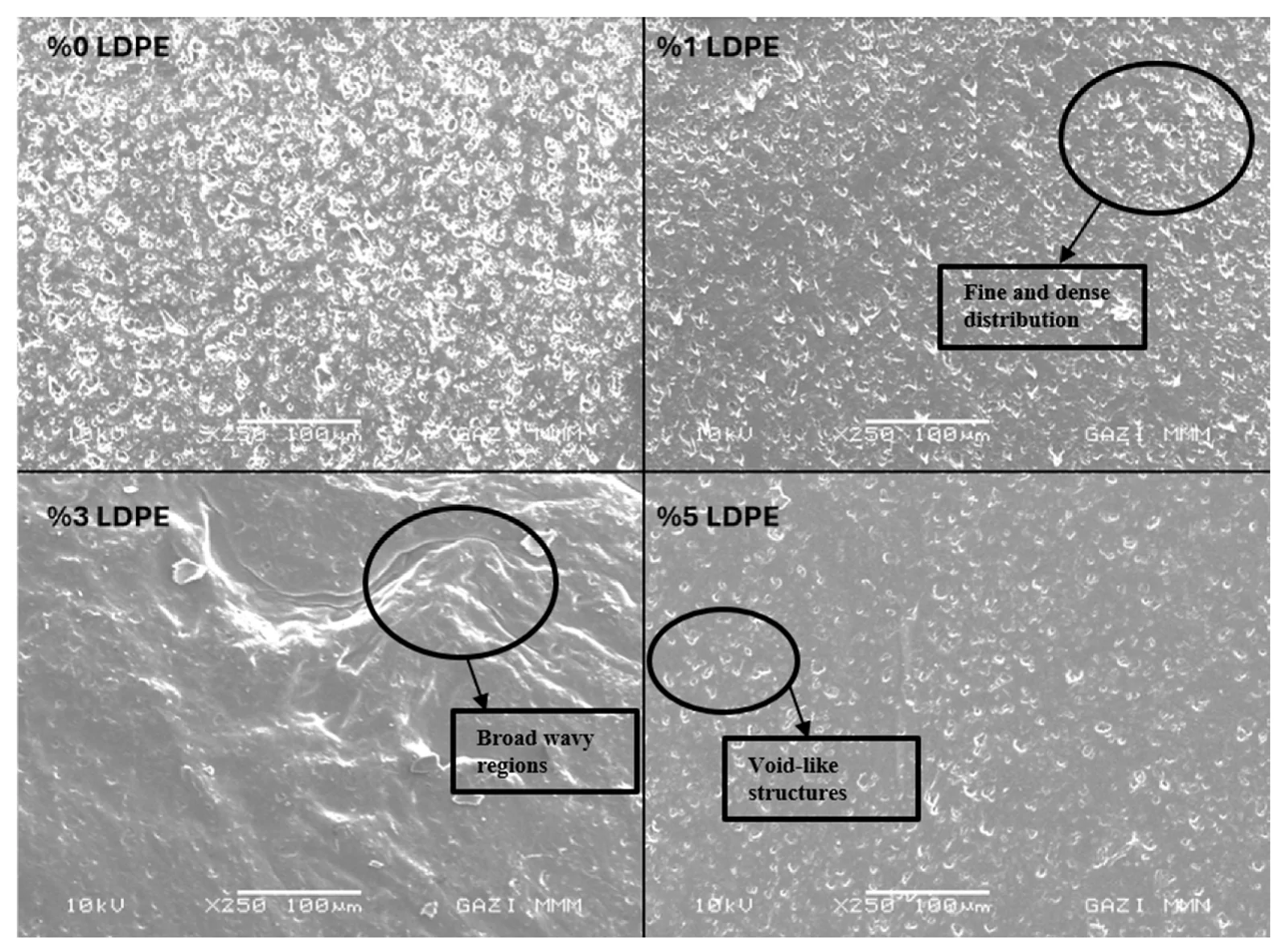
SEM images of LDPE samples with 0%, 1%, 3%, and 5% additive.

**Figure 11:**
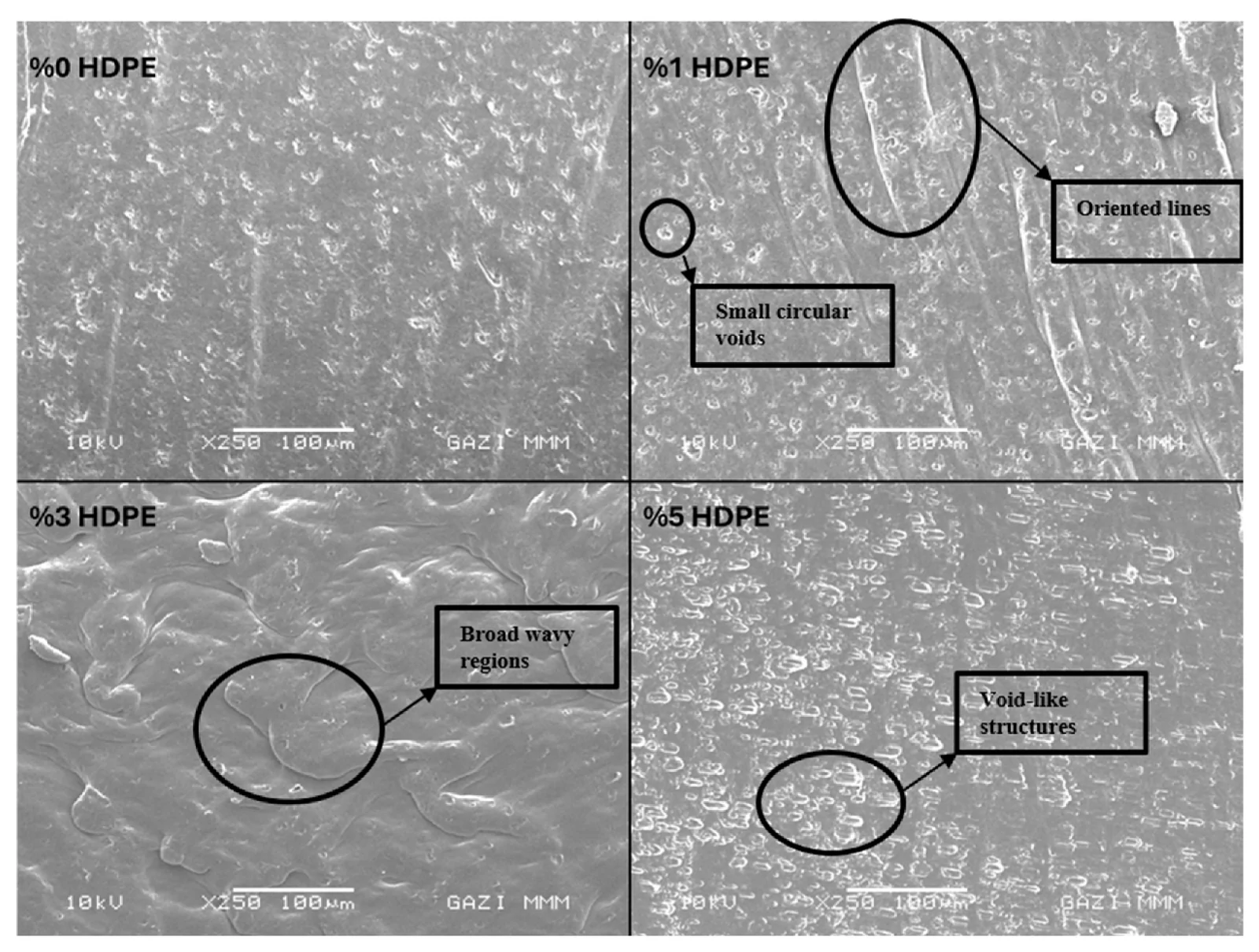
SEM images of HDPE samples with 0%, 1%, 3%, and 5% additive.

**Figure 12:**
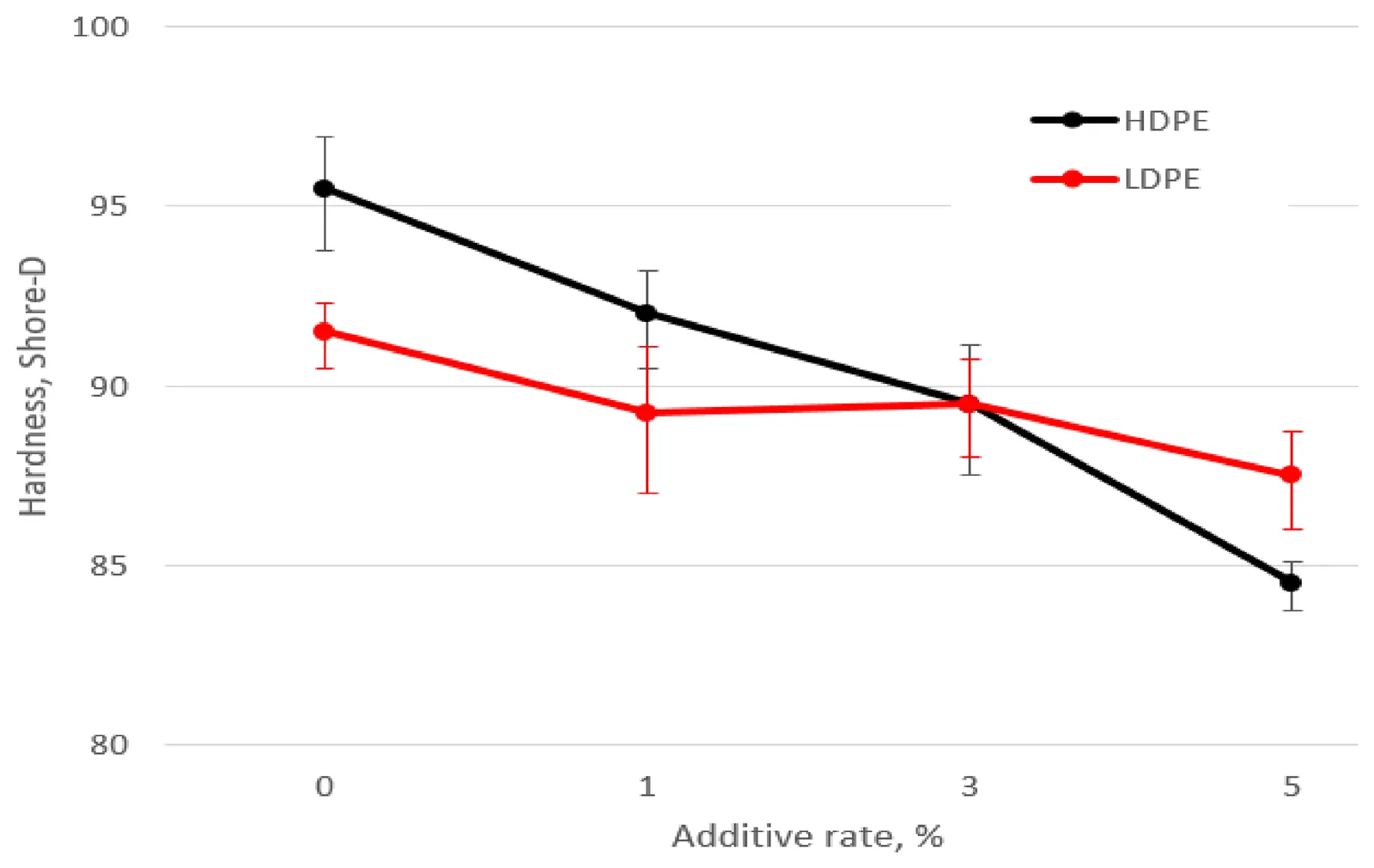
The hardness values of the HDPE and LDPE samples at different additive concentrations.

**Figure 13:**
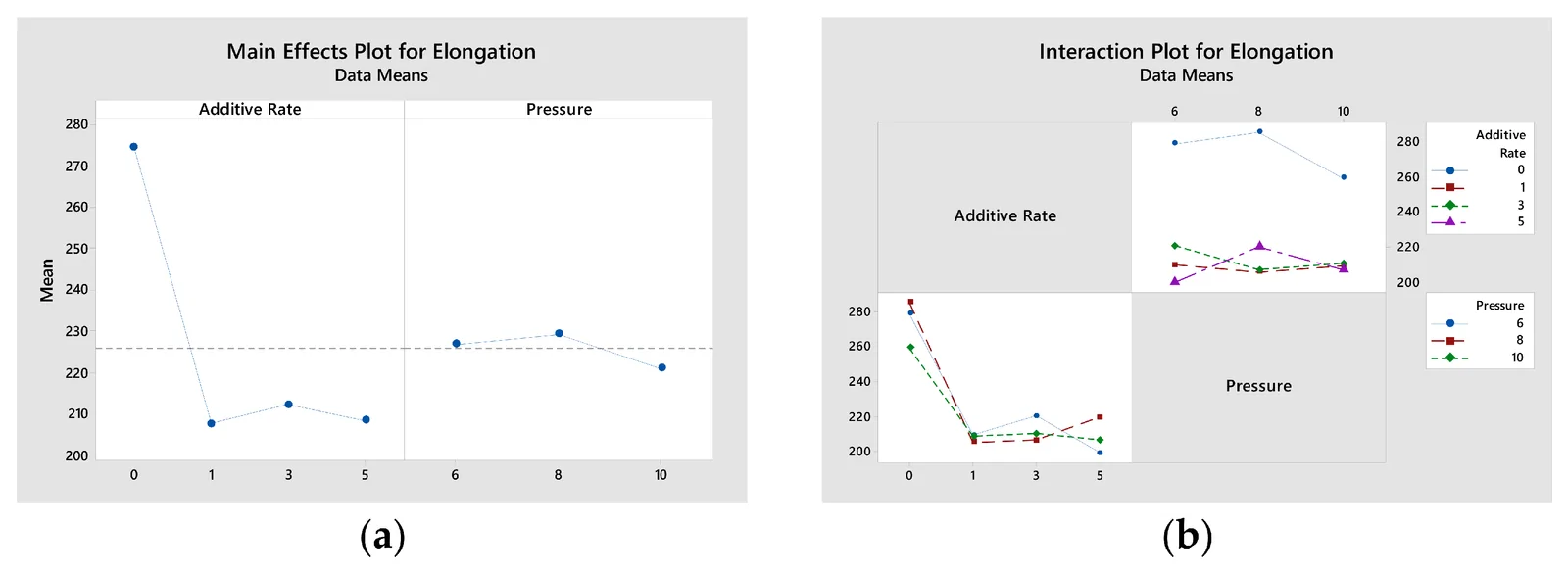
Plots of main effects (**a**) and interactions (**b**) for the elongation of LDPE material.

**Figure 14:**
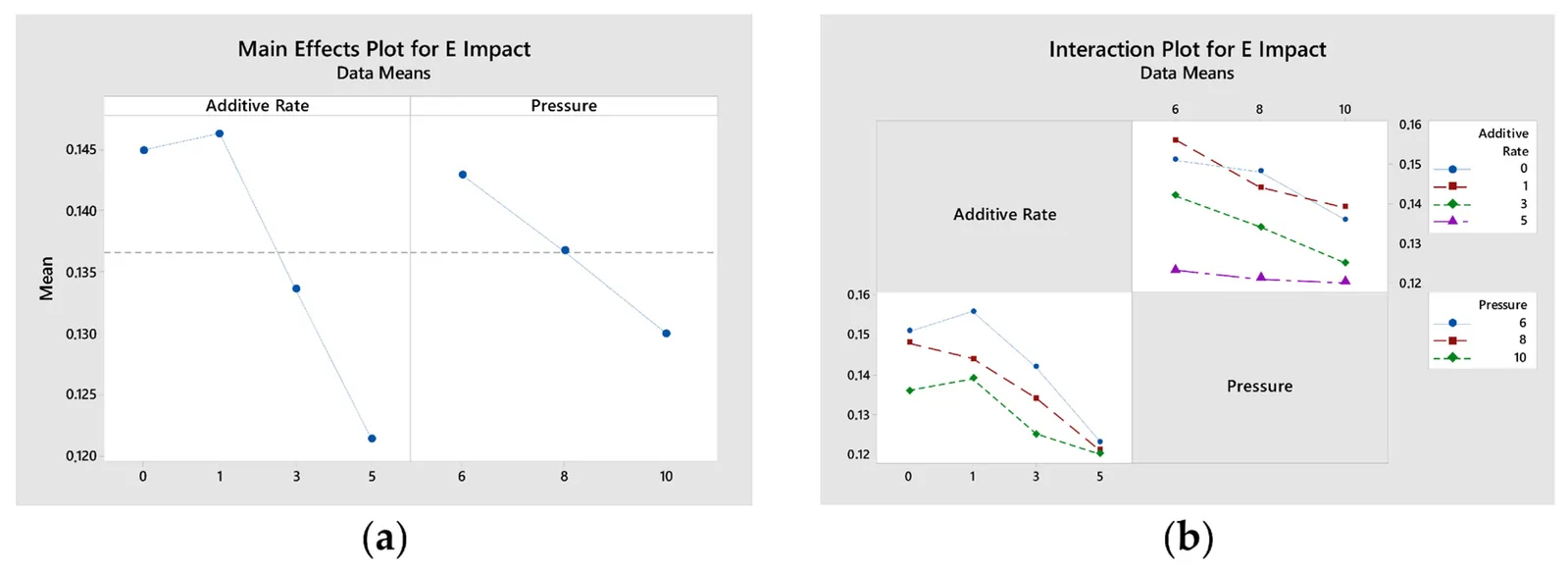
Main effects (**a**) and interaction (**b**) plots for E Impact of LDPE material.

**Figure 15:**
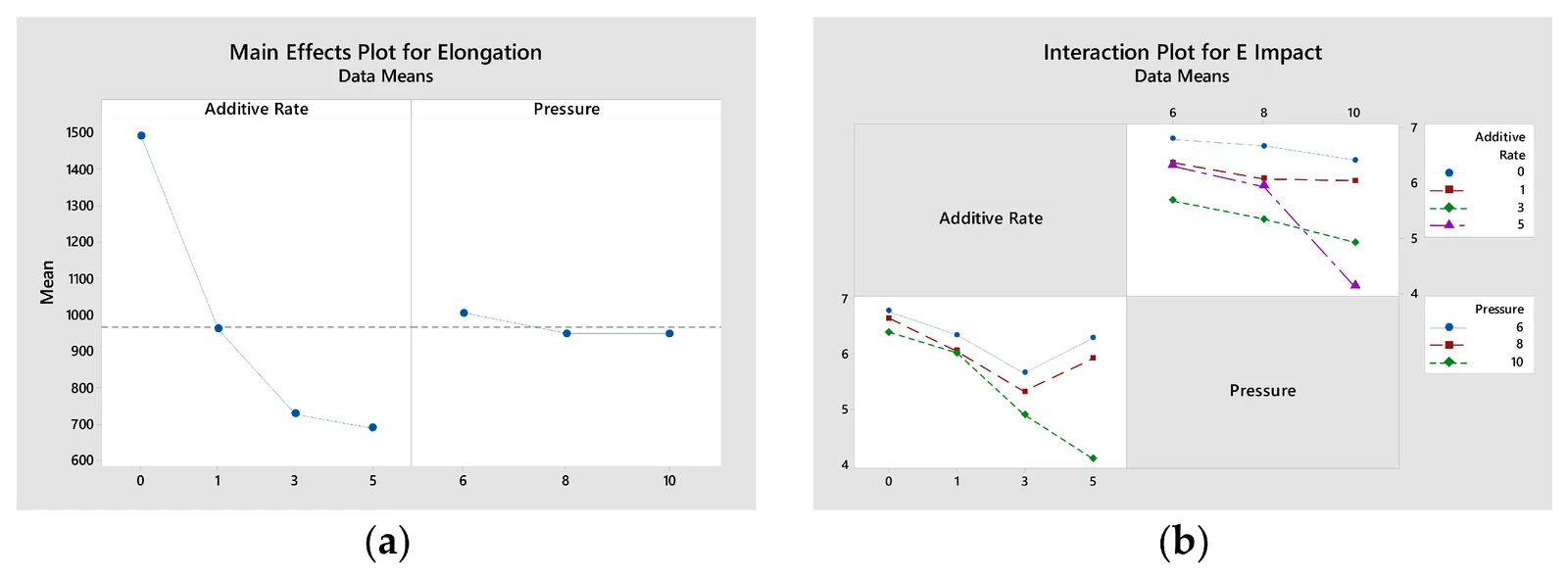
Plots of main effects (**a**) and interactions (**b**) for the elongation of HDPE material.

**Figure 16:**
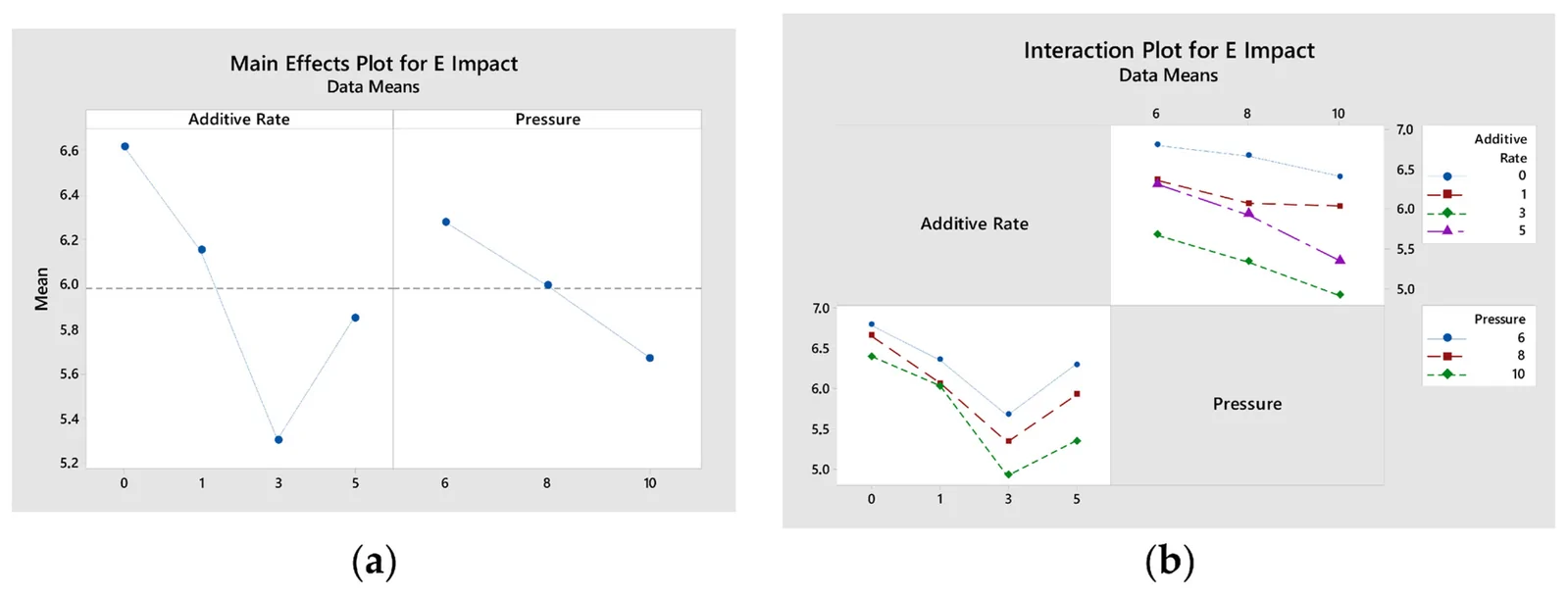
Main effects (**a**) and interaction (**b**) plots for E Impact of HDPE material.

**Table 1:** Vicat softening temperature values of LDPE and HDPE.

Material	LDPE				HDPE			
Additive content (%)	%0	%1	%3	%5	%0	%1	%3	%5
Vicat Softening Temperature (°C)	101.70	101.80	100.90	101.90	132.80	131.50	132.10	132.20

**Table 2:** Analysis of variance of LDPE plastic material for elongation.

	DF	Seq SS	Contribution	Adj SS	Adj MS	F-Value	*p*-Value
Additive Rate	3	9571	93.13%	9571	3190.32	33.88	0
Pressure	2	141.5	1.38%	141.5	70.75	0.75	0.512
Error	6	565.1	5.50%	565.1	94.18		
Total	11	10,277.5	100.00%				
Model Summary							
S	R-sq	R-sq(adj)	PRESS	R-sq(pred)			
9.70458	94.50%	89.92%	2260.29	78.01%			

**Table 3:** Analysis of variance of LDPE plastic material for E Impact.

	DF	Seq SS	Contribution	Adj SS	Adj MS	F-Value	*p*-Value
Additive Rate	3	0.001221	74.04%	0.001221	0.000407	27.18	0.001
Pressure	2	0.000338	20.51%	0.000338	0.000169	11.29	0.009
Error	6	0.00009	5.45%	0.00009	0.000015		
Total	11	0.001649	100.00%				
Model Summary							
S	R-sq	R-sq(adj)	PRESS	R-sq(pred)			
0.003869	94.55%	90.01%	0.000359	78.21%			

**Table 4:** Analysis of variance of HDPE plastic material for elongation.

	DF	Seq SS	Contribution	Adj SS	Adj MS	F-Value	*p*-Value
Additive Rate	3	1230.046	98.74%	1230.046	410.015	349.16	0
Pressure	2	8598	0.69%	8598	4299	3.66	0.091
Error	6	7046	0.57%	7046	1174		
Total	11	1245.690	100.00%				
Model Summary							
S	R-sq	R-sq(adj)	PRESS	R-sq(pred)			
34.2679	99.43%	98.96%	28182.9	97.74%			

**Table 5:** Analysis of variance of HDPE plastic material for E Impact.

	DF	Seq SS	Contribution	Adj SS	Adj MS	F-Value	*p*-Value
Additive Rate	3	2.7443	75.19%	2.7443	0.91477	34.65	0
Pressure	2	0.7471	20.47%	0.7471	0.37356	14.15	0.005
Error	6	0.1584	4.34%	0.1584	0.0264		
Total	11	3.6498	100.00%				
Model Summary							
S	R-sq	R-sq(adj)	PRESS	R-sq(pred)			
0.162484	95.66%	92.04%	0.63363	82.64%			

## Data Availability

The original contributions presented in this study are included in the article. Further inquiries can be directed to the corresponding author.
